# Proteins in Ionic Liquids: Current Status of Experiments and Simulations

**DOI:** 10.1007/s41061-017-0110-2

**Published:** 2017-02-07

**Authors:** Christian Schröder

**Affiliations:** Faculty of Chemistry, Department of Computational Biological Chemistry, University of Vienna, Vienna, Austria

**Keywords:** Direct and reversed Hofmeister series, Ionic liquid, Crystallization, Solubility, Extraction, Separation, Detection, Stabilization, Denaturation

## Abstract

In the last two decades, while searching for interesting applications of ionic liquids as potent solvents, their solvation properties and their general impact on biomolecules, and in particular on proteins, gained interest. It turned out that ionic liquids are excellent solvents for protein refolding and crystallization. Biomolecules showed increased solubilities and stabilities, both operational and thermal, in ionic liquids, which also seem to prevent self-aggregation during solubilization. Biomolecules can be immobilized, e.g. in highly viscous ionic liquids, for particular biochemical processes and can be designed to some extent by the proper choice of the ionic liquid cations and anions, which can be characterized by the Hofmeister series.

## Introduction

In contrast to common solvents, each ionic liquid intrinsically consists of two species, cations and anions, which both interact with the solute, but often in a completely different way. This offers the possibility to tune particular interactions with the solute, e.g. hydrogen bonding to one of the species, usually the anions. However, protic ionic liquid cations are also capable of forming strong hydrogen bonds [1, 2]. Cations or anions may possess long alkyl chains facilitating the penetration of a hydrophobic solute surface.

In addition to the competition to dissolve these solutes, cations and anions also interact quite strongly with each other via Coulomb forces due to their ionic nature. The exchange of the cationic and/or the anionic species or their modification results in drastic changes of the physico-chemical properties [3–5] such as the viscosity from 20 mPa s to several thousands of mPa s. Even while keeping the cationic or anionic species fixed and varying only the other, a large range of physico-chemical properties may still be accessible. However, most ionic liquids share low vapor pressures, low flammability, and significant thermal conductivity.

The interplay between ions gets even more complicated when mixing the ionic liquid with other liquids. By far, the most frequently used co-solvent is water due to its abundance, non-toxicity and biological relevance. However, depending on the nature of the composing ions, one or two phase systems with water are formed. Moreover, even one phase mixtures may be heterogeneous as ionic liquids are known to form micelles [6–9] or microemulsions [8–11]. The present review concentrates on the effects of the ionic liquid ions, whereas the consequences for water play only a minor role here. Excellent reviews focussing on water are Refs. [12–14].

## From the Ionic Liquids Point of View

### Polarity and Hydrophobicity

Chemical intuition tries to understand solvent effects and miscibility in terms of solvent polarity. According to Reichardt, polarity is defined as the overall solvation capability for molecules, including specific and non-specific interactions [15]. This rather general definition is the reason why single parameter polarity scales are often insufficient to map all interactions promoting or prohibiting solvation on a single value. Consequently, some solvation aspects are described in one solvation scale better than in the others [15, 16].

The non-specific electrostatic interactions in a liquid can be measured by dielectric spectroscopy [17]. The static (low frequency) value of the generalized dielectric constant $\varSigma _0(0)$ measures the polarization of the liquid, i.e. the total dipole moment per volume. From the amplitude of dielectric peaks it is possible to estimate the molecular dipole moment of the corresponding species. Molecules with higher dipole moments are believed to behave more polar. The high frequency limit of the dielectric constant $\varSigma _0(\omega \rightarrow \infty ) = \epsilon (\infty )$ depends on the refractive index of the liquid and hence on the polarizability per volume. Altogether, polar liquids tend to have higher dielectric constants $\varSigma _0(0)$ than apolar liquids and the corresponding values for ionic liquids are comparable to ethanol or acetone [18, 19]. Dielectric constants of aqueous mixtures depends on the occupied volume of bulk water, pressure-retarded osmosis water, and the ionic liquid [20].

**Fig. 1:**
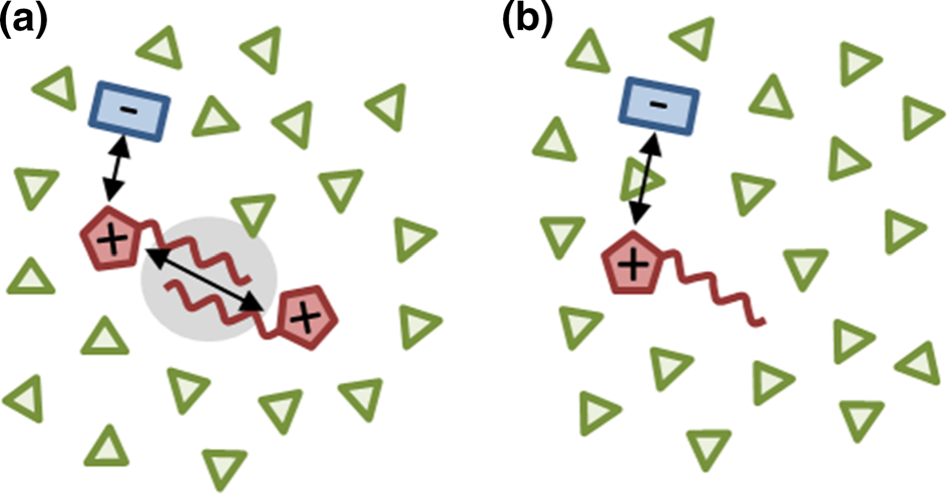
Ionic liquids in aqueous mixtures (cations in *red*, anions in *blue,* and water molecules in *green*): **a** ion aggregates and **b** solvent shared ion pairs. The *arrows* indicate strong Coulomb interactions and the *gray area* shows strong dispersion forces

The imaginary part of the dielectric spectrum of liquids with charged molecules is dominated by the static conductivity $\sigma (0)$ and, therefore, always reported in publications concerning dielectric spectroscopy. Many ionic liquids have lower conductivities than expected from their diffusion coefficients. However, this is rather due to the collective interactions of all ionic liquid ions with the other ions than to the existence of neutral ion pairs. For example, non-neutral ion aggregates (see Fig. 1a) also reduce the number of charge carriers and consequently the conductivity. These aggregates with more than two ions are already possible at moderate concentrations for simple, atomic, and small inorganic ions [14]. For example, at 1 mol L$^{-1}$, 20–30% of the ions in an aqueous solution of K[SCN] form cluster with an average size of two or three anions [14]. As ions tend to be less homogeneously distributed in ionic liquid mixtures compared to these simple ions, the concentration threshold is even lower in ionic liquids.

As depicted in Fig. 1a, clusters involving several contacts of cations are quite common even for short alkyl chains [2, 14, 21]. The non-specific dispersion interaction (gray area) between the alkyl chains is in this case stronger than the Coulombic repulsion (denoted by the black arrow). Of course, strong Coulombic attractions exist between cations and anions (also denoted by the corresponding arrow). In aqueous mixtures, water interacts with the ions and hence decreases the Coulomb attraction between the ions. However, this weakening does not necessitate that water molecules become interstitial [22] but dielectric measurements revealed that solvent assisted ion pairs (see Fig. 1b) are much more common than direct contact ion pairs/aggregates [12, 17, 23, 24]. The “solvent assistance” was explained by Robinson and Harned as “localized hydrolysis” $\ominus \cdots H^{\delta +} \cdot OH^{\delta -} \cdots \oplus$ [12, 25].

Based on solvatochromic studies another single-value scale of the polarity are $E_T^N$-values by Reichardt and co-workers [15, 26], which also characterize ionic liquids as polar as lower alcohols [15, 26–28]. A complementary polarity scale is offered by the Kamlet–Taft parameters [29]: here, dipolarity parameter ($\pi _{\mathrm{KT}}$) reflects the polarity of the solvent. It is measured by transition from ground to excited state of various dyes. As these states are stabilized by solvent dipoles and polarizabilities, the measured fluorescence shift should correlate with the solvent polarity. However, $\pi _{\mathrm{KT}}$ strongly depends on the nature of the dye and is, therefore, of reduced relevance. In addition to this non-specific interaction parameter, the Kamlet–Taft scale also characterizes local, specific interactions as the Kamlet–Taft parameter $\alpha _{\mathrm{KT}}$ and $\beta _{\mathrm{KT}}$ reflect hydrogen bond donor and acceptor capabilities. The hydrogen bond acidity ($\alpha _{\mathrm{KT}}$) is mainly determined by the ionic liquid cations. Protic cations like ethylammonium have higher $\alpha _{\mathrm{KT}}$-values than aprotic cations like imidazoliums indicating better hydrogen bond donor capabilities. The hydrogen bond basicity ($\beta _{\mathrm{KT}}$) reflects the hydrogen bond acceptor capabilities. In particular, acetate and chloride based compounds excel the other ionic liquids [29] and explain to some extent the important role for cellulose treatment [30, 31].

Hydrophobicity is a more narrow concept of polarity [32] as it characterizes the absence of favorable interaction of the solvent to water. It can be measured by the partition coefficient $\log P$ in octanol/water mixtures. High $\log P$ values indicate hydrophobic solvents. For example, hexane has a $\log P$ value of 3.5. However, 1-butyl-3-methylimidazolium acetate, nitrate and hexafluorophosphate have extremely low $\log P$ values of −2.8, −2.9 and −2.4 indicating they are much more polar than ethanol, which has $\log P=-0.24$. The $\log P$ value increases with increasing cationic alkyl chain length as expected [33].

Although hydrophobic solvents are suggested to be more favorable for enzymatic reactions [34], which was also reported by [35], protein stability may decrease with rising hydrophobicity [33, 36]. Russell and co-workers [37] could not correlate $\log P$ values with the enzyme activity and argued that the anion is responsible for the reactivity, which points to nucleophilicity or hydrogen bond basicity [32].

### Anionic Hydrogen Bonding and Cationic Surfactant Effects

1-butyl-3-methylimidazolium chloride [${\text{C}_{4}}$mim]Cl dissolves cellulose [38–40] since chloride wins the hydrogen bonding competition for cellulose OH-groups versus the intramolecular hydrogen bond network. In a molecular dynamics study of 1,3-dimethylimidazolium chloride [${\text{C}_{1}}$mim]Cl, Youngs et al. also reported that hydrogen bonds of chloride to the hydroxy groups of the carbohydrate dominated the solvation interaction [41, 42]. Armstrong and co-workers showed that the high hydrogen bond basicity is a key factor to dissolve complex polar biomolecules [43, 44]. Since hydrogen bonding is the central topic of another chapter by Patricia Hunt in this book, we will restrict our discussion to aqueous mixtures of ionic liquids.

**Fig. 2:**
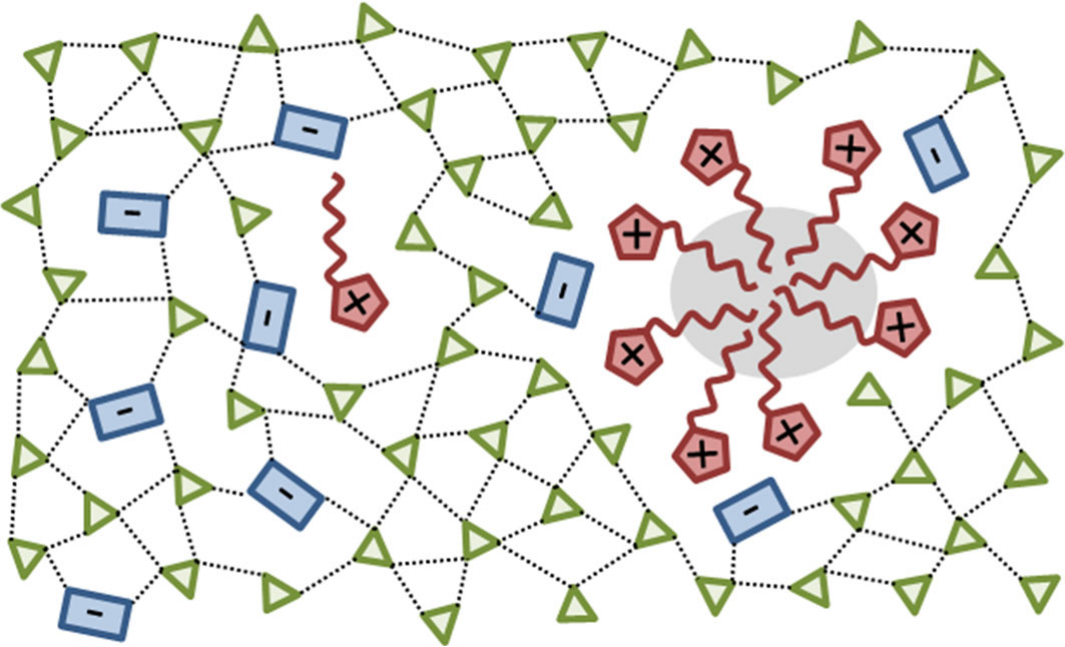
The sketch of an aqueous mixture of aprotic ionic liquids shows anion-water networks and cationic micellar-like structures

Hydrophilic ionic liquids usually consist of small anions, which can be easily implemented in the three-dimensional water network of hydrogen bonds. However, this effect of the ions is very local since femtosecond mid-IR pump-probe spectroscopy showed that only the structure of water in direct contact with the ion is influenced [45] and the effect on further solvation shells of the ion is negligible. We showed [22] that hydrophilic anions act as mediator between the more hydrophobic imidazoliums and water, which was confirmed by other authors [21, 46–49]. If the hydrogen bonded anion-water network is very strong, cations are expelled to some extent. Even at short alkyl chain lengths they may form clusters or structures similar to micelles as sketched in Fig. 2, which are held together by significant dispersion forces of the alkyl chains (gray area). Additionally, these aggregates also minimize the disturbance of the three dimensional water network because the multiple hydration of single ionic liquid cations would do much more damage to the network than the inclusion of one bigger aggregate. Such water network forces were reported by several groups [12]. The segregation into polar and apolar domains for pure ionic liquids [2, 50] and in aqueous mixtures [21] is accepted in the ionic liquid community. Consequently, the cations may accumulate at the apolar domains of proteins as well [51]. The longer the cationic alkyl chain gets, the stronger is the respective surfactant effect, which affects the stability and activity of the proteins [9, 33, 52].

### Hofmeister Series

Concerning biomolecular solvation, the so-called Hofmeister series provides a new scale to the solvation properties of ions. In 1888 Franz Hofmeister ranked several inorganic ions for their effectiveness in egg white protein precipitation. Sharing the same cation, the protein solubility increased in aqueous solutions in the following manner [53, 54]:

$$\begin{aligned} \hbox {SO}_4^{2-}<\hbox {HPO}_4^{2-}<\hbox {F}^-<\hbox {CH}_{3}\hbox {COO}^-<\hbox {Cl}^-< \hbox {Br}^-<\hbox {NO}_3^-<\hbox {I}^-<\hbox {ClO}_4^-<\hbox {SCN}^- \end{aligned}$$

However, this ranking is also used to predict protein stability in aqueous electrolyte solutions. Unfortunately, depending on the solvent conditions and the protein under investigation, the ranking is partially or even completely reversed as one may easily conduct from the plethora of rankings displayed in Ref. [55]. In order to bring some light into this confusion, we start with the underlying definitions and concepts of the Hofmeister series before jumping into its application in protein science (see Sect. 3).

**Fig. 3:**
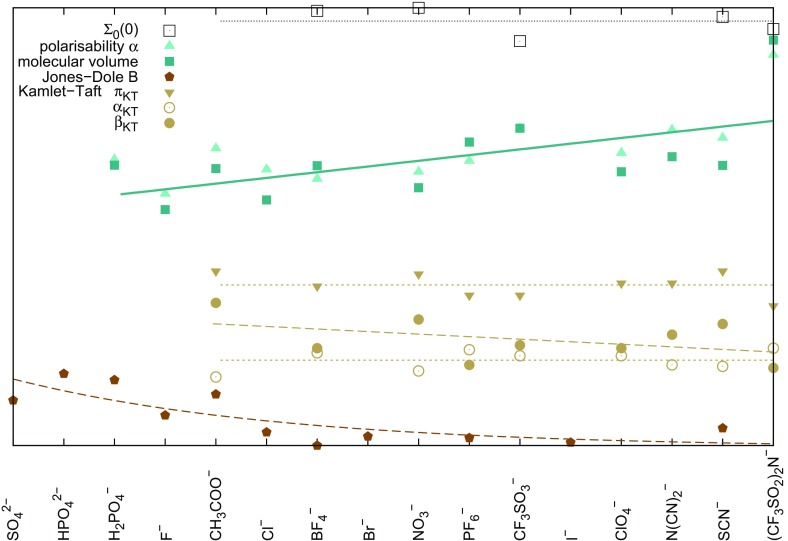
Tentative Hofmeister series (kosmotropic anions on the* left* and chaotropic anions on the* right*) including typical ionic liquid anions and their dependence on various physico-chemical descriptors (normalized and constantly shifted values). *Dashed* and *solid lines* represent decreasing and increasing trend, respectively

A tentative Hofmeister series based on the information of Refs. [54–59] is depicted in Fig. 3 using the cation 1-butyl-3-methylimidazolium. At first sight, the Kamlet–Taft dipolarity parameter $\pi _{\mathrm{KT}}$ and hydrogen bond acidity or donor capability $\alpha _{\mathrm{KT}}$ show no trend in Fig. 3, which is not astonishing as the cation usually plays the major role for these descriptors in case of ionic liquids. The decreasing Kamlet–Taft hydrogen bond accepting capability $\beta _{\mathrm{KT}}$ is also expected as kosmotropes should be more interested in hydrogen bonding than chaotropes.

Interestingly, the static dielectric constant $\varSigma _0(0)$, as well as the Reichardt’s polarity parameter $E_T^N$ (data not shown) do not systematically change from kosmotropic to chaotropic anions. In contrast, the polarizability $\alpha$ increases emphasizing the increasing importance of dispersion forces. Since the polarizability correlates with the molecular volume *V*, the same trend is also observed for *V*.

#### Kosmotropic and Chaotropic Ions

Protein stability and solubility depends on its interface with the solvent. In addition to particular interactions of single cations and anions with distinct amino acids at the protein surface, the ionic impact on protein solubility is attributed to modifying the water structure and hence the protein hydration. Ions, which strongly interact with water, are called “kosmotropic” (Greek *kosmos* $=$ order) or “structure-making”. In contrast, if the interaction with water is weak, the ions act “chaotropic” (Greek *chaos* $=$ disorder) or “structure-breaking” [60]. The basic characteristics of kosmotropes and chaotropes are summarized in Table 1. The concept of structure-making and breaking on larger dimensions was disproved by spectroscopy [45] and thermodynamic considerations [61], which showed that the influence of a central ion on the surrounding water structure is restricted to the first hydration shell and the impact on further water shells is quite small. Nevertheless, even if the effect of the ion is limited to its direct environment, the degree of hydration or hydrogen bonding may have consequences for the solvent properties. Looking at Fig. 2 again, many typical ionic liquid anions like tetrafluoroborate, triflate, or acetate show kosmotropic behavior whereas imidazoliums act more and more chaotropic when prolonging the alkyl side chains.

**Table 1:** Characteristics of kosmotropes and chaotropes adapted from [57] and including results from Fig. 3

	Kosmotropes	Chaotropes
Size	Small	Large
Hydration	Strong	Weak
Hydrogen bonds	Many	Few
Dispersion	Weak	Strong
Polarizability	Low	High
Charge density	High	Low

#### Law of Matching Water Affinities

**Fig. 4:**
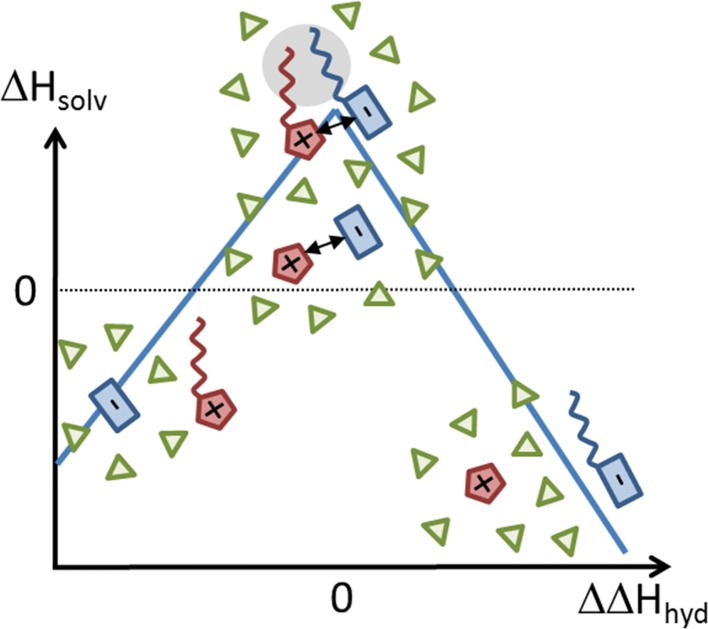
Law of matching water affinity by Collins adapted from Ref. [62] applied to the case of ionic liquids. $\Delta H_{\mathrm{solv}}$ is the enthalpy of solution and $\Delta \Delta H_{\mathrm{hyd}}$ the difference of the hydration enthalpy of the anions and the cations. Kosmotropic and chaotropic ions are depicted with and without an alkyl chain, respectively. Ion pairs in aqueous solution are only expected above the *dotted line* since their dissociation in water is favorable at negative $\Delta H_{\mathrm{solv}}$

Collins introduced the law of matching water affinities [62–64], which basically states that kosmotropic cations form ion pairs with kosmotropic anions and chaotropic cations with chaotropic anions, whereas ion pairs of mixed type do not exist, as shown in Fig. 4. In this simple volcano plot, the difference of the hydration energy of the anions and cations is correlated with the solution enthalpy of the ionic liquid. Kosmotropic anions (blue boxes without a tail) should be better dissolved in water than chaotropic cations (red pentagons with tail). As a result, $\Delta \Delta H_{\mathrm{hyd}} = \Delta H_{\mathrm{hyd}}^\ominus - \Delta H_{\mathrm{hyd}}^\oplus$ is quite negative and the dissociated pair can be found at the left part of Fig. 4. The existing Coulombic interaction between these ions is not strong enough to keep the ion pair configuration in water as visible by $\Delta H_{\mathrm{solv}} <0$. As a consequence, the anion will hydrogen bond to water, expelling the cations from the anion-water network. Kosmotropic cations and kosmotropic anions possess similar hydration enthalpies and $\Delta \Delta H_{\mathrm{hyd}} \simeq 0$. Their strong Coulombic interaction may survive in water keeping the ions together [65]. However, the strongest interaction between cations and anions exists if both are chaotropic. Here at $\Delta \Delta H_{\mathrm{hyd}}=0$, the Coulombic interaction is accompanied by strong dispersion forces. The hydrophobic tails also make these ions less attractive for water which will not penetrate the ion pair. Combining a kosmotropic ion like Li$^+$ with a chaotropic anion like octylsulfate ($\Delta \Delta H_{\mathrm{hyd}}>0$) will also result in a weak ion pair dissociating in water. However, the situation described here concerns the bulk phase of the solvent. Chaotropic ions expelled from the hydrogen bond network may act as surfactants for the dissolved protein.

#### Determination of Kosmotropic/Chaotropic Character

For simple classical ions, the kosmotropic or chaotropic character in aqueous solution can be related to the relative viscosity

1 $$\begin{aligned} \frac{\eta }{\eta _0} = 1 + A \sqrt{c} + B \cdot c \end{aligned}$$

describing the current viscosity $\eta$ with a salt concentration of *c* with respect to the pure solvent viscosity $\eta _0$ [58, 62]. The Falkenhagen *A*-coefficients depend on the electrostatics of the system and are usually small [54], whereas the *B* coefficients are ion specific and called Jones–Dole coefficients. Because of their strong interaction, kosmotropic ions hinder the motion of water and, therefore, increase the viscosity $\eta$ resulting in a positive *B* value. Chaotropic solutes should have negative *B* values. Table 2 shows some Jones–Dole *B* values of cations and anions and their kosmotropic/chaotropic assignment by Zhao [58]. Despite some minor issues concerning the anions, the assignment is quite questionable for ionic liquid cations. Many cations with long alkyl chain are claimed to be kosmotropic because of their positive *B* values. Although the high viscosities may be partially due to hydrophobic hydration [58] (and hence some interaction between the cation and water), much more importantly the viscosity rises because of adding a more viscous ionic liquid to the more fluid water. A significant source of the increased viscosity of ionic liquids are the dispersion forces in the apolar domains. Quite intuitively, prolonging the alkyl side chain should make the cation more hydrophobic and consequently less interactive with water but maybe more interactive with hydrophobic protein surfaces [53]. However, since many authors use the kosmotropic/chaotropic assignment based on *B* values, their cationic Hofmeister series shows a reversed effect [54, 55, 57]. We will stick to the simple characterization in Table 1 (as also suggested by Collins [62]) for further discussion here and concentrate on the effect of the anions in order to avoid confusion.

**Table 2:**
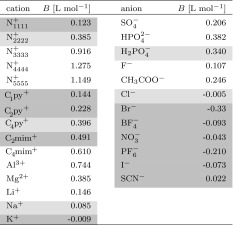
Experimental Jones–Dole *B*-coefficients [58]

The degree of gray shading imply a stronger chaotropic character based on the considerations by Zhao. The values of the imidazoliums are calculated on the basis of an empirical equation instead of a measurement

#### Impact of Ion Concentration

Regardless of the definition of kosmotropy/chaotropy, the Hofmeister ranking characterizing the protein solubility faces another problem: in 1932, Green [66] reported that the solubility of proteins in the presence of added salts, *S*, and without salt, $S_0$, followed the ionic strength *I* in a bell-shaped manner

2 $$\begin{aligned} \log \frac{S}{S_0} = \frac{1}{2} \frac{z_1 \cdot z_2 \sqrt{I}}{1+A \sqrt{I}} - K_s \cdot I \end{aligned}$$

with the ion valences $z_1$ and $z_2$ and a characteristic salt coefficient $K_s$. Although $K_s$ seems to increase with the ion volume [53] and hence chaotropic ions are expected to decrease the protein solubility, an actual Hofmeister ranking of ions depends on the ion concentration as the maximum solubility in a particular electrolyte shifts for each investigated salt. Therefore, ions may switch their position in the Hofmeister series when changing the salt concentration.

**Fig. 5:**
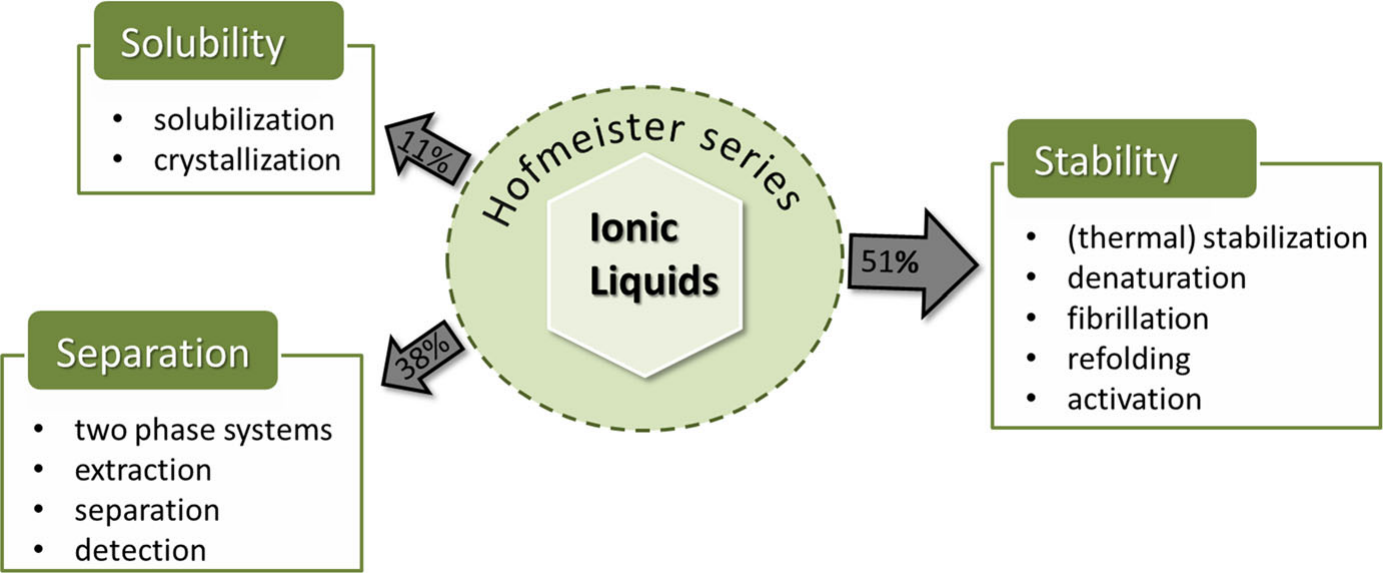
Protein applications of ionic liquids and their percentage of publications in the last decade (adapted from Refs. [69, 70])

## Through the Eyes of the Protein 

Despite all the issues concerning the “correct” ranking of the ions, the concept of a Hofmeister series is often used in the literature and has proven to be valuable to explain trends for the interaction with proteins [54, 55, 67, 68] in various fields of applications as sketched in Fig. 5 and discussed in the following sections. Wang and co-workers [70] pointed out that this particular research field is quite young, but is attracting enormous interest in chemical, food, and pharmaceutical industries. Although not taking into account all publications concerning the interactions of ionic liquids with proteins they concluded that the overwhelming number of publications (see Fig. 5) deal with protein stability in the various ionic liquid environments. Ionic liquids are able to promote or prevent denaturation (which may result in (un-)wanted fibrillation of the protein). It is also possible to activate enzymes with ionic liquids for particular reactions. Other scientific areas concern the solubility (which may be used for the crystallization) and the separation of proteins (from extraction to their detection). All these topics are discussed within the next sections with a special emphasis on protein peculiarities and their impact on the Hofmeister series.

### Protein Solubility

The vast majority of protein solubility studies concerns aqueous solutions or water mixtures with various co-solvents. Although it is possible to dissolve amino acids [71, 72] and protein in pure ionic liquids [73, 74], aqueous mixtures are preferred because of higher solubilities and/or increased stabilities of the biomolecule, as well as reduced costs.

**Fig. 6:**
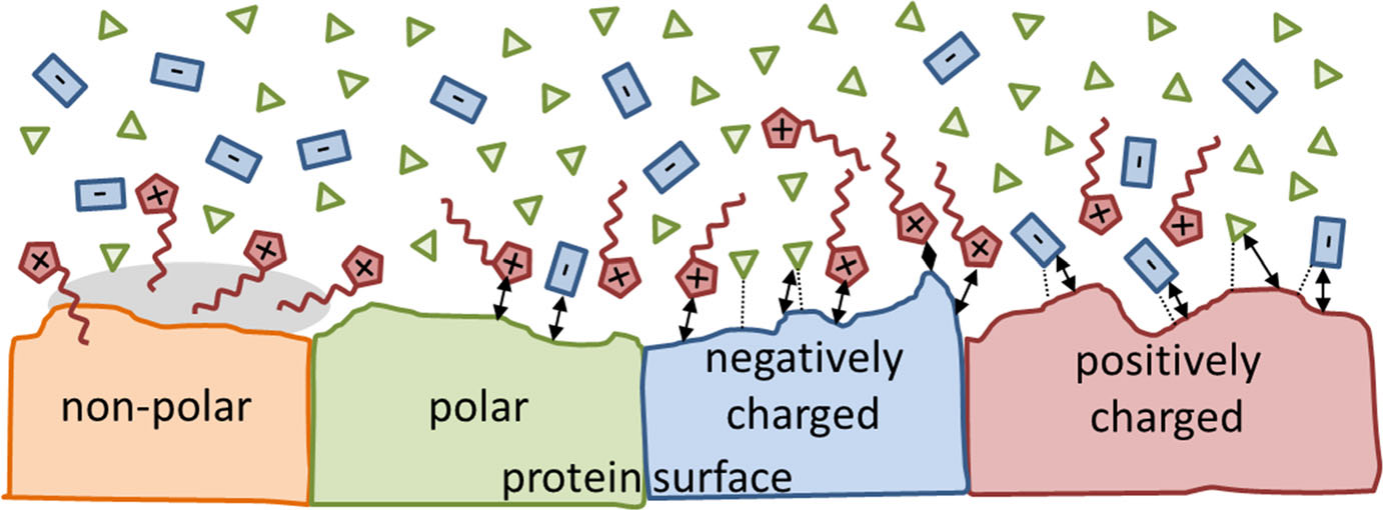
Sketch of the interactions (*dotted lines* hydrogen bonding, *gray areas* dispersion, and *arrows* strong Coulomb interaction) of the solvent species with the protein surface at higher ion concentrations (adapted from [51, 75, 76])

#### At the Protein Surface

Because of their different hydrophobic/hydrophilic/amphiphilic character, water and ionic liquid ions interact with the non-polar, polar, and charged surface areas of proteins in their own specific way resulting in an accumulation or depletion at these different regions as depicted in Fig. 6. Several simulations show that the concentration of cations exceeds that of anions at the protein surface irrespective of the protein charge [51, 75, 77–80]. This can be rationalized by the hydrogen-bonded network of the anions and water in the bulk phase (see Fig. 2 [51, 81]). Since the cations are not able to contribute to this network in the same manner, they are expelled and forced to the surface of the other hydrogen bond network violator, i.e. the protein.

Another protein solvation aspect is the strong amphiphilic character of most ionic liquid cations. Long alkyl chains attached to the “charge center” offer apolar regions with increased dispersion interactions (gray areas in Fig. 6), which are of significant importance [53, 82]. Here, hydrophobic solutes, like apolar amino acids, may find a favorable interaction partner. Klähn and co-workers reported that the alkyl chains prefer to point towards the non-polar protein surface in this case [83]. Water molecules arrange themselves in a quasi-crystalline structure (often termed “iceberg”). As a result, this hydrophobic solvation decreases the water entropy at the surface of the protein compared to bulk water [60]. However, “iceberg” models have been questioned by several groups (see [13] for details).

Since the cations are also a charged species, they compete with the anions for favorable solvation sites at the polar protein surface. The majority of cationic alkyl chains now point away from the protein surface [83]. Of course, the negatively charged amino acids attract more cations than anions, but they still are in competition with water molecules (green triangles in Fig. 6), which may form hydrogen bonds to the surface amino acids in contrast to the cations. The approach of cations to the positively charged protein surface is not excluded per se since cationic charge densities are low and is also enabled by the close-by anions at the protein surface.

The (hydrophilic) anions seem to prefer the positively charged surface as they try to establish hydrogen bonds to lysine, arginine, or histidine (if positively charged) [44] and show strong Coulomb interaction with these amino acids due to their high charge density. Overall, the Coulombic interaction of the anions with the protein is stronger compared to the cations resulting in a longer residence time of that molecule near the respective amino acids [75, 78]. The cations are quite mobile at the non-polar and polar protein surface and only show increased residence times in the proximity of glutamic and aspartic acid. This cationic mobility may be also a reason that active protein sites are visited more by cations than anions [75].

Both cations and hydrophilic anions usually interact stronger with the protein than water and hence remove water from the protein surface with increasing ionic liquid concentration [71, 75]. Here, the impact of the anion seems to be more important [71]. However, the water removal may have positive and/or negative consequences for the protein solubility [84]. Hydrophobic ionic liquids may form two phase systems with water resulting in less depletion of water molecules from the protein surface and hence an increased protein solubility [85, 86].

#### Following the Hofmeister Series

In addition to these surface effects, the Hofmeister series discussed in the last chapter concerns the impact of the ions on the bulk water structure that influences the water structure at the protein surface. A significant correlation between water-water hydrogen bonding and the experimental solubility data for hydrophobic solutes was found in computer simulations [87] as expected by the Hofmeister effect. Quite generally, the term “salting in” refers to the effect at moderate ion concentrations up to $1\,\hbox {mol}\,\hbox {L}^{-1}$ that increasing ionic strength of a solution enhances the solubility of the proteins. Looking at the Hofmeister series, the protein solubility increases with the chaotropic character of the anions [82, 88] and results in an opposite “salting-out” effect for strong kosmotropic anions like phosphate and sulfate [53, 82]. MD simulations [89] showed that the reduction of hydrophobic interactions leads to salting-in and may be entropic and/or enthalpic whereas the salting-out induced by kosmotropes is purely an enthalpic effect. However, one has to keep in mind that “salting-in” and “salting-out” also depend on the nature of the protein [60, 90, 91] since they are not homogeneous in charge, hydrophilicity/hydrophobicity, as well as secondary and ternary structure [53]. Netz and co-workers pointed out that the Hofmeister prediction is (more or less) valid for negatively charged proteins whereas the reversed order may be more appropriate for neutral or positively charged proteins [90, 91]. This reversed Hofmeister series seems to be “the rule rather than the exception” for the small inorganic cations and anions [91].

#### Protein Crystallization

However, high protein solubilities are not always desirable since (more or less) controlled precipitation and protein crystallization in good quality are worthwhile for X-ray structure determinations [92]. Usually, at high ion concentrations, the protein solubility drops significantly and the protein precipitates. This process can be used for the separation of proteins to get purer crystals since the necessary salt concentration depends on the nature of the protein. In particular, due to their manifold and “tunability” ionic liquids attracted interest as co-solvents for protein precipitation and crystallization since 1999 when Garlitz and co-workers reported on lysozyme crystallization modified by ethylammonium nitrate [93]. Judge et al. reported that various proteins (lysozyme, catalase, myoglobin, trypsin, glucose isomerase, and xylanase) grow crystals of larger size with ionic liquids as co-solvent, which provide a better X-ray diffraction resolution [92]. However, the impact of ionic liquids on the crystallization process is more pronounced than for precipitation [92, 94]. Several authors report on less crystal polymorphism and improved tolerance to concomitant impurities during crystallization [94–99].

Peter Nockemann and co-workers noticed that increasing the concentration of the ionic liquid (regardless of the Hofmeister character) results in a reduction of crystal nucleation density and improved crystal quality [76]. Choline based ionic liquids showed less efficiency than imidazolium based ionic liquids and prolonging the cationic alkyl chain length in the imidazolium salts improved the efficiency. However, the concentration dependence observed by Green [66] and discussed in the Hofmeister Sect. 2.3.4 was also observed for ionic liquids [76]. Electrostatic forces play the major role at low ion concentrations and the specific impact of the ion correlates with their screening of the protein surface charges to reduce the repulsion between like-charged biomolecular regions and hence promotes protein aggregation. He also pointed out that the dehydration of the anions is important for the binding to the protein surface, i.e. the anion-water network in Fig. 2 has to be overcome making kosmotropic anions less effective. Cations may have the opposite effect since the binding to non-polar residues (cf. Fig. 6) counteracts the previously discussed effect and reduce the interfacial tension promoting protein-solubility. As a result, ions, which bind to the protein surface and screen the surface charges at low concentration promoting salting-out, induce salting-in at higher concentrations by remaining hydrated at the protein surface.

### Separation of Proteins

The separation of target protein accounts for 50–80% of its total production costs [100, 101] and the tunability of ionic liquids has been exploited in this context [100–108]. Also, the recovery and purification of enzymes from bioreaction media gained importance because of a increasing demand for biotechnologically manufactured fine chemicals and biomolecules [105].

In principle, the pure ionic liquid or its mixture with water may be used to dissolve the proteins. However, in pure ionic liquids most proteins are dispersed, but not homogeneously dissolved [102, 107, 109, 110]. Furthermore, pure ionic liquids may denaturate the protein (which will be discussed in the next Sect. 3.3) as they need water to maintain their natural structure and function.

Aqueous mixtures of ionic liquids can also be decomposed in “hydrated ionic liquids” (high ion concentrations) and electrolyte like solutions (low ion concentrations), which have different solvation properties for a particular protein. Furthermore, these solutions may also change their solvation behavior as a function of temperature offering a nice route to extraction and separation by precipitation [107].

#### Two-Phase Systems

The largest area of ionic liquid application for extraction and separation of proteins are two-phase systems: Of course, hydrophobic ionic liquids form two phases with water. However, these hydrophobic ionic liquids are usually more expensive and viscous than hydrophilic ionic liquids and may denature proteins [106]. Rogers and co-workers [111] were the first to report on two-phase systems with hydrophilic ionic liquids at certain concentrations overcoming the limitations mentioned above. The aqueous phase usually contains $\hbox {K}_2\hbox {HPO}_4$. The kosmotropic anion interacts stronger with water than the interaction between water molecules. The water-hydrogen bond network is therefore enhanced resulting in a stronger expelling of the cations (see Fig. 2). Various salts including $\hbox {K}_3\hbox {PO}_4$, $\hbox {K}_2\hbox {CO}_3$, $\hbox {K}_2\hbox {SO}_4$, and others also have been tested [112] but $\hbox {K}_2\hbox {HPO}_4$ combined a high solubility in water with an excellent ability to promote phase separation with the ionic liquid. These two-phase systems are distinguished by their protein selectivity, robustness, short processing time, low energy consumption and easy scale up opportunities [108, 113]. Cation [101] and anion [104] effects have been studied by the Coutinho group. As expected, long alkyl chains attached to imidazoliums promote phase separation and partitioning [100, 101]. Inserting double bonds, benzyl or hydroxyl groups leads to less efficiency. Kragl and co-workers [105] reported a strong correlation between the protein charge and the partition behavior and suggested that electrostatic interactions at the protein surface with the cations are the major driving force of protein partitioning in the two phases. The efficiency of the two phase system also follows the Hofmeister series for the anions [104, 106].

The extraction efficiency seems to increase with increasing temperature indicating an endothermic process [100]. The enthalpic $\Delta H^0_{\mathrm{ILphase}}$ and entropic change $\Delta S^0_{\mathrm{ILphase}}$ associated with the protein partitioning measured by the Gibbs energy $\Delta G^0_{\mathrm{ILphase}}$ is obtained from the partitioning coefficient *K*

3 $$\begin{aligned} \Delta G^0_{\mathrm{ILphase}} = - R T \ln K = \Delta H^0_{\mathrm{ILphase}} - T \Delta S^0_{\mathrm{ILphase}} \end{aligned}$$

as a function of temperature. Both $\Delta H^0_{\mathrm{ILphase}}$ and $\Delta S^0_{\mathrm{ILphase}}$ are positive for the transition of the protein bovine serum albumin from the $\hbox {K}_2\hbox {HPO}_4$ to the ionic liquid rich phase. However, the overall $\Delta G^0_{\mathrm{ILphase}}$ is negative since $T \Delta S^0_{\mathrm{ILphase}}$ exceeds $\Delta H^0_{\mathrm{ILphase}}$. The importance of entropic effects indicate the major role of hydrophobic interactions [100, 108].

#### Protein Detection

However, ionic liquids are not only advantageous for extraction and separation, but also for the detection of particular proteins. Traditional capillary electrophoresis for protein separation results in broadened bands and low protein recovery. Dynamic coating of the capillary with imidazolium-based ionic liquids suppress protein adsorption and generates an anodic electroosmotic flow [114–116]. A recent review [117] summarizes the current status of capillary electrophoresis. Furthermore, the vacuum stability of ionic liquids and their solvation properties is also beneficial for ionic liquid matrices in matrix-assisted laser desorption/ionization mass spectrometry (MALDI) [118]. Particularly, ionic liquid matrices promote sample homogeneity, increase ion peak intensities, and lower detection limits compared to conventional solid matrices [119].

### Protein Stability

Proteins are a particularly heterogeneous class of biological macromolecules. For their functioning, it is very important to maintain their secondary structure, i.e. $\alpha$-helices, $\beta$-sheets, and coil regions. These structural elements are held together via a complex balance of hydrogen bonds, disulfide bridges, hydrophobic and ionic interactions. In most of these interactions solvent molecules participate or compete with the involved amino acids. Consequently, changing solvent conditions like viscosity, pH value, buffer conditions, addition of (ionic) co-solvents, and temperature has a severe impact on the secondary structure of the protein. The influence of solvent viscosity differs from the other solvent conditions since higher viscosities decelerate the overall dynamics of the solvent and the dissolved protein, mimicking higher protein stability within the observed time window [16, 77, 120]. However, the viscosity $\eta$ is a central solvent property.

#### Gibbs Free Energy of Unfolding

The pH value of the solvent and the presence of ions are important for the Coulomb interactions of the solvent with the protein. The preferred protonation state of the amino acids within the protein changes significantly with the pH and the buffer conditions thereby changing the character of the local protein surface from polar to charged or back (changing also the protein preference for solvent molecules depicted in Fig. 6). As a function of ion concentration, these amino acids will have stronger or weaker interactions with the solvent contributing to stabilization enthalpy. The temperature, on the other hand, and the exchange of water, cations, and anions at the surface of the protein govern the entropic contributions.

In fact, native protein structure is only marginally stable as visible by the slightly positive Gibbs free energy of unfolding [68]

4 $$\begin{aligned} \Delta G_{\mathrm{unfolding}} = \Delta H_{\mathrm{unfolding}} - T \cdot \Delta S_{\mathrm{unfolding}} \end{aligned}$$

describing the transition from the native to an unfolded state. The low value of $\Delta G_{\mathrm{unfolding}}$ is based on the mutual compensation of significant enthalphic ($\Delta H_{\mathrm{unfolding}}$) and entropic ($\Delta S_{\mathrm{unfolding}}$) contributions [68], which can be shifted by adding co-solvents like ionic liquids. For example, choline dihydrogen phosphate stabilizes cytochrome c [110, 121] and lysozyme [122] for months. Brogan and Hallett [73] reported on the freeze-drying properties of 1-butyl-1-methylpyrrolidinium salts. Overall, the protein storage lifetime ranges from a few days to more than a year depending on the nature of the protein and its environment [110, 123, 124].

**Fig. 7:**
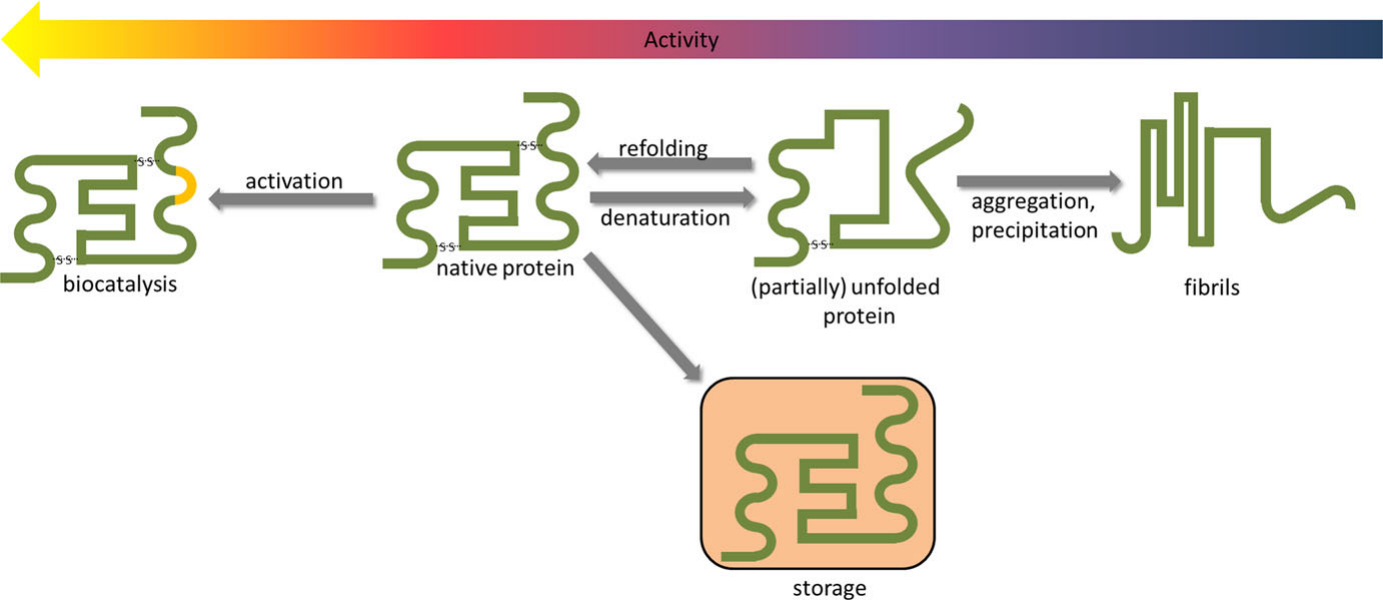
The protein activity depends on secondary structure. Ionic liquids may be used to activate particular amino acids, to store proteins for longer time periods, to help refolding and to prevent aggregation

Ionic liquids may shift the subtle balance of $\Delta H_{\mathrm{unfolding}}$ and $\Delta S_{\mathrm{unfolding}}$ in one or the other direction resulting in denaturing [56, 125–131] or refolding/stabilizing of the protein [33, 109, 123, 126, 132–135]. In principle, the native protein structure and the (partially) unfolded state of the protein are in a reversible equilibrium (Fig. 7, [124, 134, 136]). This unfolding/refolding equilibrium is disturbed by an irreversible protein aggregation followed by fibrillation which completely deactivates the protein. The protein aggregation can be hindered by ionic liquid as co-solvents [132, 137, 138] protecting the hydrophobic parts of the protein surface. The cations seem to cover these hydrophobic areas and suppress interprotein accumulation.

However, ionic liquids can also be applied to enhance the activity of the native protein. This area of applications is reviewed elsewhere [16, 32, 57, 113] in more detail and only briefly summarized here: they may chemically modify the protein or stabilize a (genetic) protein modification or immobilize the protein at a surface for chemical reactions [32]. Because of their tunable properties, particular ionic liquids are capable to selectively solubilize reactions and/or products, which offers in two-phase systems better product separation and improved recoverability of the catalyzing protein (as discussed in the Sect. 3.2). Ionic liquids also influence the enantioselectivity of reactions [55].

In contrast to classical polar organic solvents some polar ionic liquids seem to activate particular proteins [32, 113, 120, 139], whereas the activity of another enzymes is diminished [32, 37, 113, 140]. One has to bear in mind that reaction rates in different ionic liquids are usually compared at the same amount of water. Under such conditions, solvents with higher polarity would have less water associated with the protein probably reducing the reaction rate. On the other side, the increased water content in the bulk phase reduces the viscosity and thereby increases the protein mobility [141] and hence the activity. Depending on the importance of these factors for the protein, the reaction rate increases or drops.

#### Computer Simulations

There are several experimental techniques to study the protein conformation like UV-vis, fluorescence, IR, Raman, and NMR spectroscopy, circular dichroism, tensiometry, small-angle neutron scattering, differential scanning calorimetry, and microcalorimetry are listed in [69] and references therein. Since experiments monitor only the overall effect of the ionic liquids on these topics of protein activity, protein simulations are quite useful for the interpretation of the role of particular cations and anions. The current state of the art was recently reviewed by Shaw [142]. In principle, the protein stability can be followed in molecular dynamics computer simulations in various ways:

1. Monitoring the van der Waals and Coulomb interactions
  5 $$\begin{aligned} U = \sum \limits _i \sum \limits _j 4 \epsilon _{ij} \left( \left( \frac{\sigma _{ij}}{r_{ij}} \right) ^{12} - \left( \frac{\sigma _{ij}}{r_{ij}} \right) ^6 \right) + \sum \limits _i \sum \limits _j \frac{q_i \cdot q_j}{4 \pi \epsilon _0 r_{ij}} \end{aligned}$$
  between the protein and the solvent or within the protein. If the atom *i* is part of a protein and the atom *j* belongs to a solvent molecule, the sum above represents the protein–solvent interactions [75]. In this case, major contributions of the van der Waals part commonly stem from the interaction of the amphiphilic ionic liquid cations with the protein, whereas very strong Coulombic interactions between the ionic liquid anion and positively charged amino acids can be found.
  Steinhauser and co-workers [77] computed the van der Waals and Coulomb interaction within the protein as a function of the mole fraction of water in the aqueous 1-ethyl-3-methylimidazolium triflate mixture. With increasing water content, the Coulomb interaction rises whereas the van der Waals interaction decreases, which was explained by a transition from dipolar screening to charge screening and its consequences on the solvation structure. Dipolar screening describes the reduction of Coulomb attraction and repulsion by interstitial water molecules, which is quite effective due to the high dielectric constant of water. The ionic liquid ions screen the protein surface charge since these interactions are usually stronger than the attraction or repulsion between neighboring amino acids. Interestingly, the most unfavorable *U* was found at a mole fraction $x_{\mathrm{H_2O}}$ of 0.93, which also coincides with the lowest conservation of secondary structure.
2. Programs like DSSP [143] assign secondary structure elements to each amino acid based on the current protein coordinates from the trajectory. This way, the extension or shrinking of $\alpha$-helices and $\beta$-strands can be followed during simulations and compared to the native protein structure. Over the complete trajectory averaged stability values were compared for several mole fractions in Ref. [77] also detecting the lowest protein stability at $x_{\mathrm{H_2O}}=0.93$, which indicates that the stability of ubiquitin and the zinc finger is not a monotonic function of the solvent viscosity.
3. The transient root-mean-square deviation ${\mathrm {RMSD}}(t)$ of a protein is defined as
  6 $$\begin{aligned} {\mathrm {RMSD}}t) = \sqrt{\frac{\sum \nolimits _{i} \Bigl (\mathbf {r}^{\mathrm{ref}}_i-\mathbf {r}_i(t)\Bigl )^2}{N}} \end{aligned}$$
  using the current coordinates of the *i*th protein atom $\mathbf {r}_i(t)$ and those values of a reference state $\mathbf {r}^{\mathrm{ref}}_i$, which is usually the protein in its native configuration. If the ${\mathrm {RMSD}}(t)$ is monitored for longer time periods one should also consider the protein rotation before applying Eq. 6.
  Growing ${\mathrm {RMSD}}(t)$ indicate that the protein moves away from its native state. It is also possible to compute ${\mathrm {RMSD}}(t)$ for particular secondary structure elements of a protein. Thus, the stability of particular $\alpha$-helices and $\beta$-sheets can be characterized. For example, the increase of ${\mathrm {RMSD}}(t)$ of $\alpha$-helices in serine protease cutinase when adding [${\text{C}_{4}}$mim][PF$_6$] or [${\text{C}_{4}}$mim][NO$_3$] was interpreted by Soares et al. [81] as an attack of the ions on the secondary structure. Klähn and co-workers [79] also detected by RMSD less stable $\alpha$-helices compared to $\beta$-sheets with the same ionic liquids but in the lipase *Candida antarctica B*.
4. The radius of gyration $R_\mathrm{g}(t)$ is also an indicator: since unfolding goes along with an increase of the protein volume, the loss of secondary structure leads to an increase of the radius of gyration
  7 $$\begin{aligned} R_\mathrm{g}(t) = \sqrt{\frac{\sum _i m_i (\mathbf {r}_i(t) - \mathbf {r}_{\mathrm{CM}}t))^2}{M}} \end{aligned}$$
  as the protein loses its compactness and increase the average distance of the protein atom *i* to the proteins center-of-mass $\mathbf {r}_{\mathrm{CM}}(t)$. The sum of each atomic mass $m_i$ is the mass of the protein *M*. Klähn [79] observed that the radius of gyration, the RMSD and the stability of $\alpha$-helices and $\beta$-sheets followed the same trend for the investigated ionic liquids.
5. The effect of (partial) unfolding is even more prominent in the solvent accessible surface since coil regions are much more accessible by the solvent molecules than $\alpha$-helices and $\beta$-strands. In Ref. [79] changes of the solvent accessible surface area coincide with the stability criteria mentioned above. However, for the zinc finger in [77] the correlation between the surface accessible area and the protein stability is detectable but not very pronounced.

All these methods aim for the (more or less) collective effect of the solvent at a mesoscopic level. The next section deals with the impact of the ionic liquid ions at the molecular level.

#### The Impact of the Ionic Liquid Ions

Weingärtner et al. [136] ranked typical ionic liquid ions in a Hofmeister series for the *ribonuclease A* stability in the following order:

$$\begin{aligned} \hbox {N}_{1111}^+ || \hbox {chol}^+> \hbox {N}_{2222}^+ \simeq \hbox {C}_{2}\text{mim}^+ \simeq \hbox {gua}^+> \hbox {C}_{4}\text{{C}}_{1}\text{py}^+> \hbox {C}_{4}\text{mim}^+ \simeq \hbox {N}_{3333}^+ > \hbox {C}_{6}\text{mim}^+ \simeq \hbox {N}_{4444}^+ \end{aligned}$$

with $\hbox {chol}^+$ and $\hbox {gua}^+$ being choline and guadinium respectively and

$$\begin{aligned} \hbox {SO}_4^{2-}\!\!>\hbox {H}_2\hbox {PO}_4^-\!\!>\hbox {CH}_3\hbox {COO}^-\!\!>\hbox {Cl}^- || \hbox {EtSO}_4^-\!\!>\hbox {BF}_4^-\!\!>\hbox {OTf}^-\!\!>\hbox {SCN}^-\!\!\simeq \hbox {N(CN)}_2^- \gg \hbox {NTf}_2^- \end{aligned}$$

Ions on the left side of || stabilize ribonuclease, whereas the others destabilize the protein. This finding corresponds to classical Hofmeister behavior that kosmotropic ions increase and chaotropic ions decrease the protein stability [82, 144]. However, other authors argued that proteins are more stable in hydrophobic ionic liquids [75, 79, 109, 124, 145] since they keep the protein water layer intact [79, 81, 120]. The supporters of the direct Hofmeister series displayed above argue that the protein solubility in hydrophobic ionic liquids is very low and the observed stabilization of protein refers to finely dispersed proteins in a heterogeneous solvent [136].

**Table 3:** Ion specific affinities for surfaces with different functional groups [91]

Functional group	Anion binding affinity	Cation binding affinity
Hydrophobic $\hbox {CH}_3$	Reversed Hofmeister	Direct Hofmeister
Polar $\hbox {OH}^-$	Direct Hofmeister	Inconclusive
Polar COOH	Direct Hofmeister	Direct Hofmeister
Charged $\hbox {COO}^-$	Direct Hofmeister	Reversed Hofmeister

**Fig. 8:**
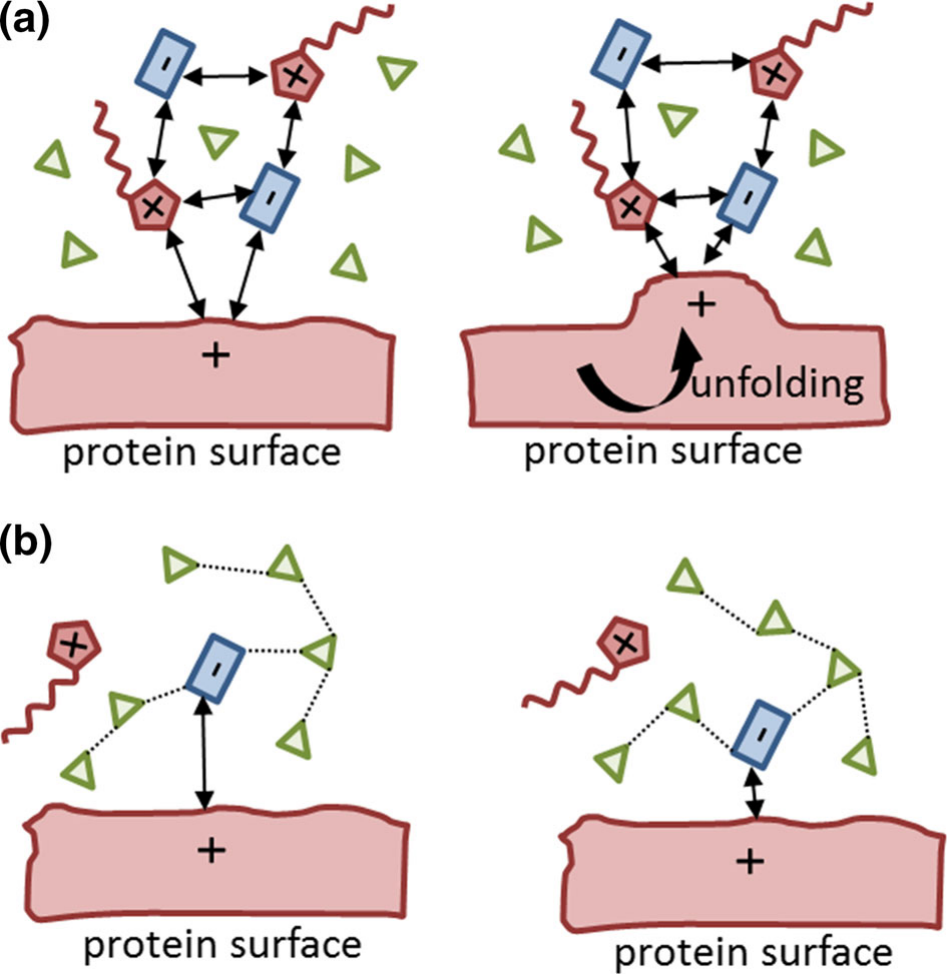
**a** Unfolding mechanism proposed by Ref. [79], **b** alternative interpretation of the strong interaction of the anion with the protein surface

The central role of the anions was pointed out by several computational studies [77–79, 81]. Although the concentration of cations seems to be usually higher than that of the anions [51, 77–79], the mean residence time of the anion at the surface [77] is much longer due to the strong Coulomb and hydrogen bonding interaction sketched in Fig. 6. The affinity trends to particular functional groups were analyzed for classical inorganic anions in Ref. [91] and is tabulated in Table 3. Although typical ionic liquid ions were not investigated in [91] the trends should still hold. This may have positive and/or negative consequences for the unfolding/refolding equilibrium. Klähn et al. [79] pointed out that the interaction of cations and anions with a positively charged protein surface is enhanced during the unfolding process as sketched in Fig. 8a. They assume that the ionic liquid ions prefer to approach the protein surface pairwise due to their strong cation-anion network. The Coulomb repulsion of the cation with the protein surface is overcompensated by the attraction of the anion to the surface (left picture of Fig. 8a). As a consequence, the positively charged amino acid moves towards the anion starting the unfolding process depicted in the right figure of Fig. 8a). Although the repulsion to the cation gets stronger, the increased attraction to the anion promotes this step. However, as discussed for Fig. 2, neutral cation-anion clusters are not favorable, in particular for hydrophilic anions which prefer hydrogen bonding to water [22, 46]. Consequently, the “anion clusters” possess less mass than an ion pair. If the anion is attracted to the protein surface, it will move towards the surface (see Fig. 8b). On the other hand, the positively charged amino acid moves not very much towards the solvent phase in contrast to Fig. 8a.

**Fig. 9:**
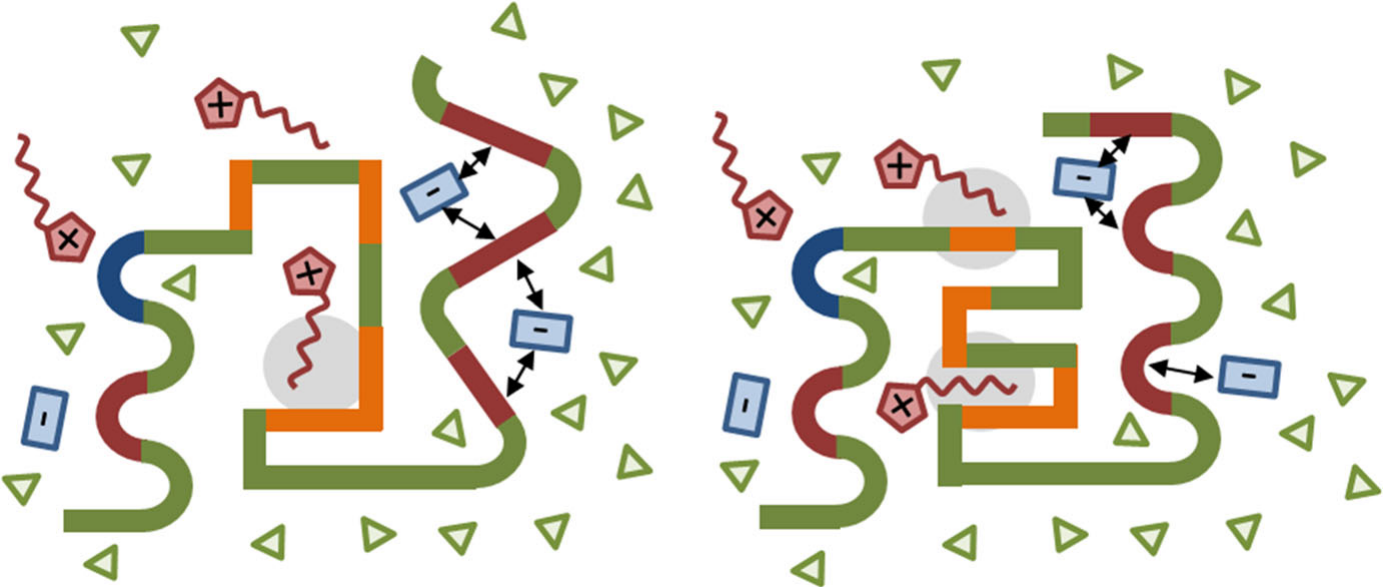
Adapted refolding mechanism of Ref. [132]. *Blue*, *red*, *green*, and *orange areas* reflect negatively charged, positively charged, polar and non-polar amino acid sequences. The *arrows* denote strong Coulomb interaction and the *gray shaded area* strong dispersion

Strong interaction between the anion and the positively charged amino acids may also lead to refolding as depicted in Fig. 9 and suggested by Summers and Flowers [132]. Because of the strong interaction of an anion with several positively charged amino acids, these amino acids are forced into a more compact structure facilitating the building of hydrogen bonds necessary for the secondary structure elements, i.e. $\alpha$-helices and $\beta$-strands. The structure compressing in case of apolar amino acids can be promoted by the apolar regions of the amphiphilic cations. However, more computational studies investigating various ionic liquid ions and their interaction on particular amino acids including the consequences on the secondary structure are necessary to finalize the picture of the ionic liquid impact on the protein structure.

#### Thermodynamics of the Hofmeister Series

Ebbinghaus and co-workers reported that the protein stability is also a function of the ion concentration [68]: At low ion concentration ($c_{\mathrm{IL}}< 0.5\,\hbox {mol}\,\hbox {L}^{-1}$) almost all aqueous ionic liquid mixtures denature proteins. At higher ionic liquid concentration above $1\,\hbox {mol}\,\hbox {L}^{-1}$ ion-specific effect becomes dominant and the Hofmeister series more meaningful [68]. Therefore, the concentration behavior of stabilizing and destabilizing ionic liquids is different as measured by the salt-induced shift of the protein melting temperature $\Delta T_m$. At $c_{\mathrm{IL}}=0\,\hbox {mol}\,\hbox {L}^{-1}$ the shift starts at $\Delta T_m=0\,\hbox {K}$. Adding small amounts of the ionic liquid destabilize the protein resulting in negative $\Delta T_m$-values for all ionic liquids. However, in stabilizing ionic liquids $\Delta T_m$ increases with increasing concentration reaching values of more than 20 K for choline dihydrogenphosphate at $c_{\mathrm{IL}}=4\,\hbox {mol}\,\hbox {L}^{-1}$ [68]. In destabilizing ionic liquids, $\Delta T_m$ always decreases with increasing ion concentration reaching values of $\Delta T_m=-25 \hbox {K}$ for $[\hbox {C}_{2}\text{mim}]$[SCN] at $c_{\mathrm{IL}}=1.5\,\hbox {mol}\,\hbox {L}^{-1}$. Interestingly, $\Delta T_m$ directly correlates with $\Delta \Delta G_{\mathrm{unfolding}}$ defined by

8 $$\begin{aligned} \Delta \Delta G_{\mathrm{unfolding}}&=  \; \Delta G_{\mathrm{unfolding}}^{\mathrm{IL}} - \Delta G_{\mathrm{unfolding}}^{\mathrm{buffer}}\end{aligned}$$

9 $$\begin{aligned}&= \; \Delta \Delta H_{\mathrm{unfolding}} - T \cdot \Delta \Delta S_{\mathrm{unfolding}} \end{aligned}$$

Both contributions in Eq. 9 are very ion specific and follow the direct Hofmeister series or its reversed order as visible in Table 4. For example, $\Delta \Delta S_{\mathrm{unfolding}}$ follows the direct Hofmeister series for the cations whereas the reversed ranking is observed for $\Delta \Delta H_{\mathrm{unfolding}}$.

**Table 4:** Trends of the generalized Hofmeister behavior [68, 91] at salt concentrations of $1\,\hbox {mol}\,\hbox {L}^{-1}$

Cations	
Protein surface $\oplus$	Reversed Hofmeister
Protein surface $\ominus$	Direct Hofmeister
$\Delta T_m$	Reversed Hofmeister
$\Delta \Delta G_{\mathrm{unfolding}}$	Reversed Hofmeister
$\Delta \Delta H_{\mathrm{unfolding}}$	Reversed Hofmeister
$T \cdot \Delta \Delta S_{\mathrm{unfolding}}$	Direct Hofmeister
Anions	
Protein surface $\oplus$	Reversed Hofmeister
Protein surface $\ominus$	Direct Hofmeister
$\Delta T_m$	Direct Hofmeister
$\Delta \Delta G_{\mathrm{unfolding}}$	Direct Hofmeister
$\Delta \Delta H_{\mathrm{unfolding}}$	Inconclusive
$T \cdot \Delta \Delta S_{\mathrm{unfolding}}$	Inconclusive

This has several consequences:

- based on the sign of $\Delta \Delta S_{\mathrm{unfolding}}$ the mechanism to stabilize the protein may be primarily enthalpic or entropic.
- because of the common enthalpy-entropy compensation at room temperature, varying the temperature changes the stabilizing/denaturing property of the ionic co-solvent. In other words, the Hofmeister series is temperature dependent.
- since the enthalpic and entropic contribution depend on the pH of the solution and protein charge, isoelectric point, and hydrophobicity, sometimes the overall impact of the ions neither follow the direct nor the reversed Hofmeister series.

**Fig. 10:**
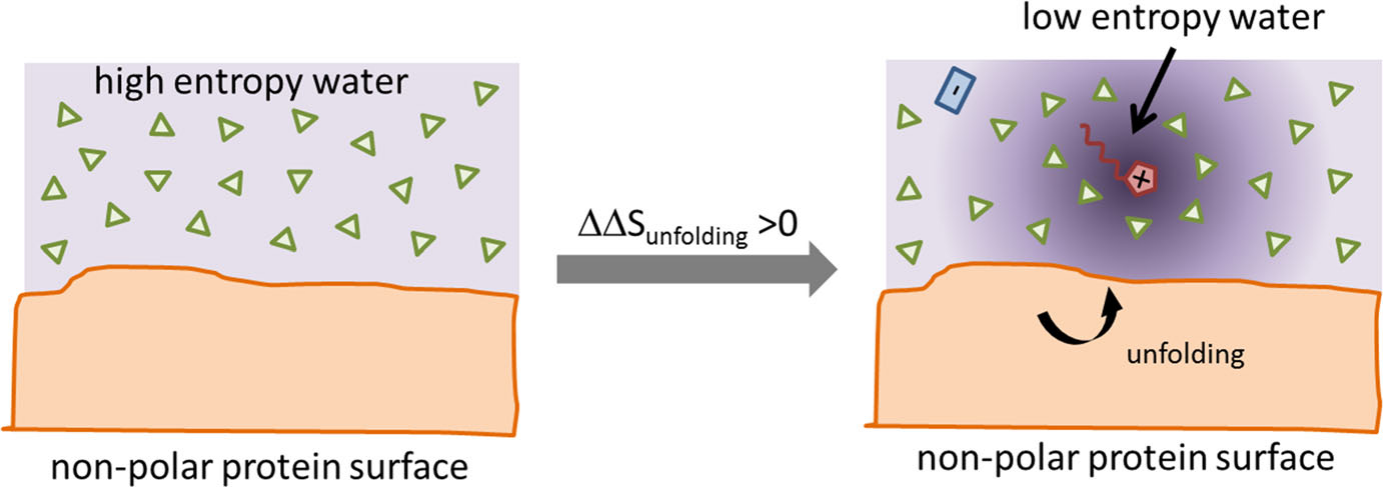
Hydrophobic solvation of hydrophobic co-solutes reduces the water entropy and promotes unfolding since the penalty due to the solvation of the hydrophobic residues is less severe. This figure is adapted from [68]

However, stabilizing enthalpic contribution ($\Delta \Delta H_{\mathrm{unfolding}}>0$) and the counteracting entropic contribution ($T \cdot \Delta \Delta S_{\mathrm{unfolding}}>0$) tend to rise with increasing hydrophobicity of the ionic liquid cation. If the water entropy is reduced by the hydrophobic co-solute, the entropy decrease due to unfolding and hence solvation of hydrophobic protein residues becomes less important and is, therefore, promoted by the hydrophobic co-solute as visible in Fig. 10.

## Conclusion

Many appealing solvation properties of ionic liquids and their tunability by proper choice of the cation and anion combination have been investigated in various areas of biochemistry in the past two decades. The findings for the protein solubility, crystallization, separation, and stability are often mapped on the Hofmeister series or its reversed order.

Unfortunately, the involved interactions and effects compensate each other to a large extent. Consequently, the overall effect of a particular ionic liquid on a protein cannot be predicted and necessitates further investigation from experimenter and computational scientist. In particular, it would be useful to apply a reasonable set of the very same ionic liquids to several areas of protein research depicted in Fig. 5. So far, the comparison and the deeper understanding of the underlying mechanisms is hampered by the fact that majority of publications use very special ionic liquids or reaction conditions, which do not allow for generalizations.
